# Case report: CD19-directed CAR-T cell therapy combined with BTK inhibitor and PD-1 antibody against secondary central nervous system lymphoma

**DOI:** 10.3389/fimmu.2022.983934

**Published:** 2022-10-05

**Authors:** Wenqi Zhang, Chen Huang, Ruixia Liu, Huichao Zhang, Weijing Li, Shaoning Yin, Lianjing Wang, Wei Liu, Lihong Liu

**Affiliations:** ^1^ Department of Hematology, The Fourth Hospital of Hebei Medical University, Shijiazhuang, China; ^2^ Hebei Provincial Key Laboratory of Tumor Microenvironment and Drug Resistance, Shijiazhuang, China; ^3^ Clinical Laboratory, The Fourth Hospital of Hebei Medical University, Shijiazhuang, China

**Keywords:** scnsl, CD19 CAR-T cell therapy, BTK inhibitor, PD-1 antibody, zanubrutinib, tislelizumab

## Abstract

Current therapeutic strategies for central nervous system (CNS) relapse of diffuse large B-cell lymphoma (DLBCL) are extremely limited. Secondary central nervous system lymphoma (SCNSL) also shows a grave prognosis and high mortality. This report describes a young female patient with DLBCL and CNS relapse who received low-dose CD19-directed chimeric antigen receptor T (CAR-T) cell therapy followed with Bruton’s tyrosine kinase inhibitor and programmed cell death protein 1 antibody after several lines of chemotherapy. However, limited reports on CAR-T cell therapy are applied for SCNSL, particularly those in combination with targeted agents. The current treatment combination for this case provides a new regimen for CNS relapse from DLBCL.

**Clinical Trial Registration:**

ClinicalTrials.gov number, NCT04666168.

## Introduction

Central nervous system (CNS) relapse is one of the most devastating complications of diffuse large B-cell lymphoma (DLBCL). Studies show that CNS relapse in aggressive non-Hodgkin’s lymphoma (NHL) patients accounts for 2%–27%, and the prognosis is poor with the median overall survival (OS) of only 3.9 months (1, 2). The existence of the blood–brain barrier (BBB) prevents immune and/or chemotherapeutic drugs from penetrating into the brain and, thus, limits the therapeutic effect (3). Currently, the primary treatments for secondary central nervous system lymphoma (SCNSL) include whole brain radiation therapy (WBRT), high-dose chemotherapy–autologous stem-cell transplantation (HDCT-ASCT), or intravenous high-dose methotrexate. CD19-directed chimeric antigen receptor T (CAR-T) cell therapy, combined with novel agents, is one of the promising paradigm-changing options for CNS lymphoma (4). In 2017, the first successful case using CD19-directed CAR-T cell therapy was reported for CNS-DLBCL (5). Investigations and studies about CAR-T cell therapy in conjunction with other novel targeted agents, including Bruton’s tyrosine kinase (BTK) inhibitor, programmed cell death protein 1 (PD-1) antibody revealed synergistic action for relapsed/refractory (R/R) DLBCL (6–8). BTK inhibitors could enhance the function and implantation of CAR-T cells (9). PD-1 antibody can promote their intracranial anticancer activity (10). However, TP53, as a tumor suppressor gene, modulates apoptosis in DNA-damaged cells and controls cell proliferation; the prognosis always becomes worse, and there is frequent chemotherapy resistance in DLBCL with this gene mutation (11). Promising treatment with an alternative mechanism, such as CAR-T cell therapy, could obtain a better prognosis than cytotoxic agents in a retrospective study observed by Edit Porpaczy et al. (12).

In this study, we report a case of DLBCL of CNS relapsed with TP53 mutation. The patient was enrolled in a clinical trial (ClinicalTrials.gov number, NCT04666168), which is a multicenter clinical study on the safety and efficacy of CAR-T cells in the treatment of R/R NHL as the third line therapy. In this case, we combined BTK inhibitor and PD-1 antibody with anti-CD19 CAR-T cell therapy. The patient achieved complete response (CR) for more than 18 months without any complication from this combination strategy and maintained an optimistic survival status.

## Case description

A 38-year-old female patient initially presented with cough and expectoration for a month. The patient showed shortness of breath and chest tightness lasting 20 days without fever and dyspnea. The CT scan showed mediastinal masses, and treatment with antibiotics and glucocorticoid was ineffective in the local hospital. The symptoms worsened, and the patient was admitted to the emergency room at the Fourth Hospital of Hebei Medical University in October 2018. The positron emission tomography/computed tomography (PET/CT) showed several soft tissue masses, and multiple lymph nodes were found in the mediastinum, right axillary, around the thyroid, at thoracic entrance level, both internal mammary regions, behind the left diaphragmatic angle, retroperitoneum, and near right iliac vessels. The maximal standardized uptake value (SUV max) was 7.9. Some masses were distributed in the mediastinum and compressed the heart. The immunohistochemistry (IHC) from the mediastinal mass revealed CD3-, CD20+, CD21-, CD30-, Ki-67(90%+), AE/AE3-, BCL-2+, BCL-6+, CD10+, CD5-, CD23-, MUM1+, CMYC(5%), and TDT-, and the Han’s algorithm was applied to determine germinal center B-cell-like (GCB) and non-GCB phenotypes according to IHC using anti-CD10, MUM1, and BCL6 antibodies (13). The result of fluorescence *in situ* hybridization (FISH) presented with EBER-. A complete blood count showed the white blood count was 10.26×10^9^/L; the red blood count was 4.47×10^12^/L; the platelet count was 339×10^9^/L and hemoglobin was 124.9g/L. The patient’s LDH and β2-microglobulin levels were 257 U/L and 1.37 mg/L, respectively. The patient did not present with fever, drenching night sweats, and loss of body weight, which were defined as “B symptoms” at initial diagnosis. Based on these examinations, the patient was diagnosed as stage III A GCB-DLBCL (international prognostic index (IPI) 3 points, KI-67 90%). Afterward, the patient received therapy with five cycles of rituximab combined with cyclophosphamide, liposomal doxorubicin, vincristine, and prednisone acetate (R-CHOP) in the General Hospital of the People’s Liberation Army (PLAGH). The patient had a history of hepatitis B, and the HBV DNA level was detected at 5.88×10^6^ IU/mL. Entecavir plus tenofovir disoproxil fumarate was used for antiviral therapy. Then, the patient obtained CR by PET/CT examination 4 months after the initial presentation. Another three cycles of R-CHOP were conducted for consolidation and maintenance treatment.

**Figure 1:**
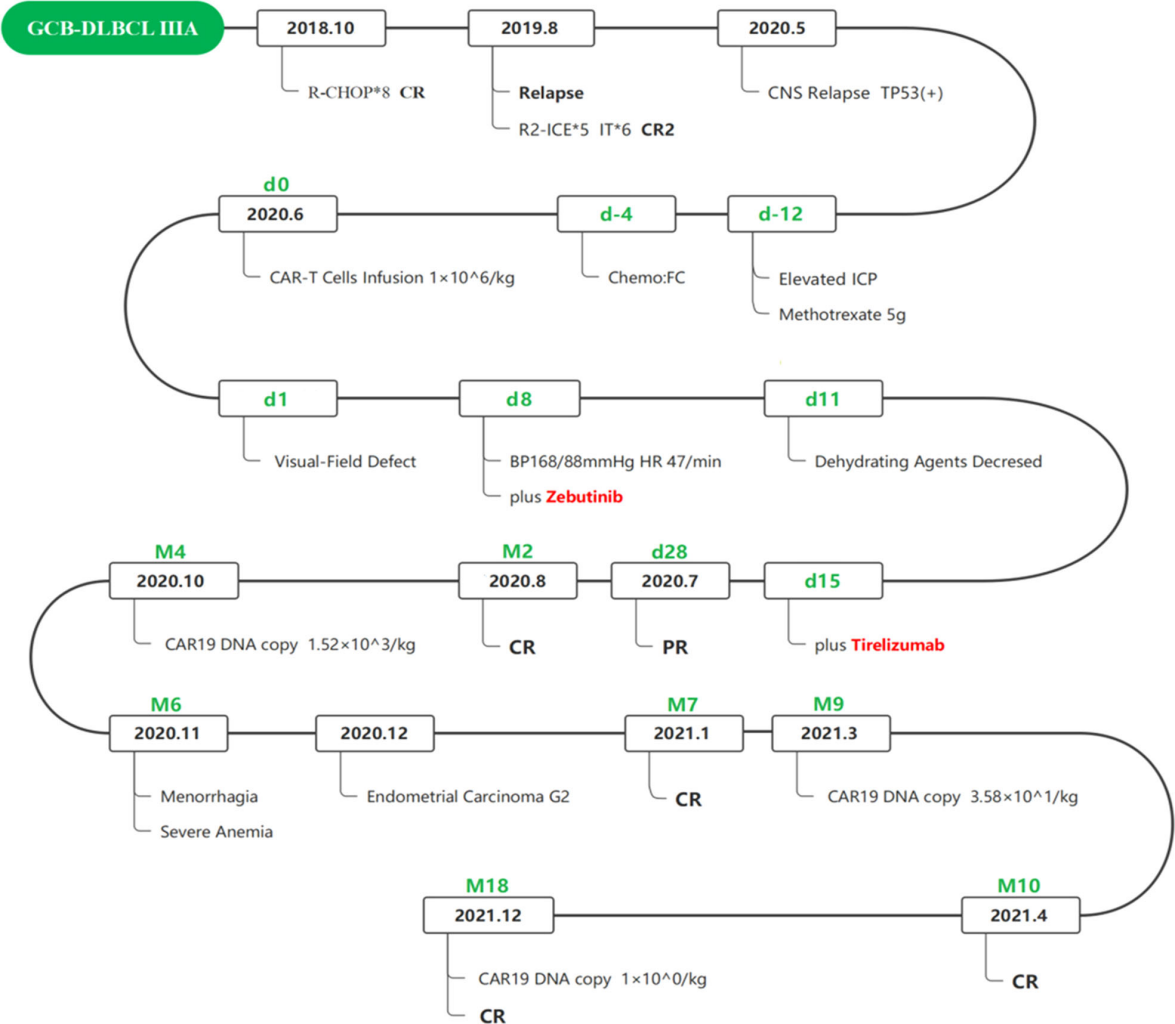
Flow chart of the disease process and therapeutic modalities.

However, the first relapse occurred 4 months after the last chemotherapy **(** **Figure 1** **)**. The patient developed lymphadenectasis in the right cervical and axillary regions and was admitted to the department of hematology in PLAGH. The PET/CT examination and CT scan indicated a large range of lesions with high metabolism, including endometrium and several parts of the bilateral diaphragm, except for the brain. The masses were considered to invade the spinal canal at T7 and T10 levels. There were also small nodules on the left breast without increased metabolism. Consequently, the patient was diagnosed with stage IV A relapse GCB-DLBCL and treated with five cycles of rituximab (600 mg on day 0) combined with ifosfamide, etoposide, carboplatin (R2-ICE), and oral lenalidomide. The mecapegfilgrastim injection was given to avoid agranulocytosis with fever after chemotherapy. Then, the patient received six cycles of methotrexate plus dexamethasone by intrathecal injection for CNS prevention. Several cerebrospinal fluid (CSF) laboratory tests revealed no abnormalities. Afterward, a PET/CT examination showed high-density enlarged lymph nodes in bilateral diaphragm regions, high metabolic parts in the endometrium, and a small nodule on the left breast disappeared. As a result, the patient was evaluated as CR2 on March 2020, but she refused consolidation chemotherapy and ASCT for further treatment.

**Figure 2:**
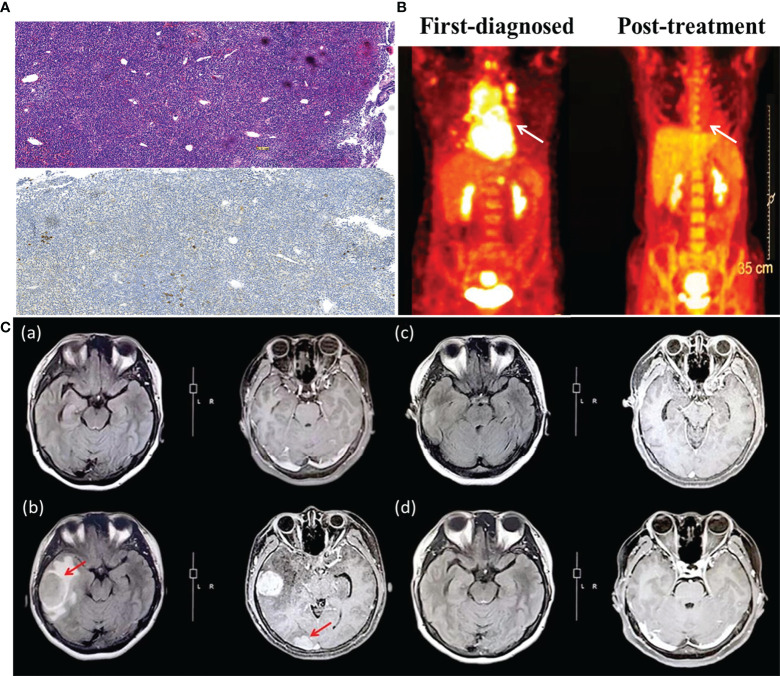
**(A)** The pathological section of mediastinum lymphatic tissue (upper panel: hematoxylin and eosin stain; lower panel: immunohistochemistry, CD30 negative). **(B)** Positron emission tomography assessment of patient first diagnosed (left) and after CAR-T cells infusion (right). The white arrow indicates the invasion of DLBCL on the mediastinum, which is the primary site. **(C)** Brain MRI images before and after CAR-T cell therapy. **(a)** Brain MRI on March 2020, after the second cycle of chemotherapy of R2-ICE*5 and evaluated as CR2. **(b)** Brain MRI on May 2020, when the patient showed CNS relapse. The size of the right temporal lobe and occipital lobe masses are 3.19 cm×4.03 cm and1.75 cm×2.4 cm, respectively. **(c)** The same region on October 2020, more than 4 months after CAR-T cells infusion. The mass almost disappeared and was evaluated as CR for 2 months. **(d)** The follow-up image on December 2020 with continuous CR.

Approximately 2 months later, the patient disease relapsed for the second time (**Figure 1**). The patient felt a headache for a week and was admitted to the department of hematology in the Fourth Hospital of Hebei Medical University. The compression in the right temporal and occipital lobes presented by cranial contrast-enhanced magnetic resonance imaging (MRI), and intracranial tumor infiltration was considered. The biopsy was performed by stereotactic surgery under general anesthesia. A right temporal lobe mass IHC revealed CD3-, CD20+, CD19+, CD21-, CD30-, KI-67(80%+), CD10+, C-MYC(2%), BCL-2+, BCL-6+, MUM1+, CD38-, CD5-, and CyclinD1- (**Figure 2A**) and negative EBER by FISH test. Next-generation sequencing was conducted, which revealed the presence of a TP53 mutation (**Table 1**). Consequently, the patient was diagnosed with stage IV A CNS relapse GCB-DLBCL (TP53+), which implicated a poor prognosis.

**Table 1:** Gene mutation result from NGS report. The 55 hot spot genes of DLBCL were screened.

TP53	NM_000546:exon7:c.T687A :p.C229X	82.28%	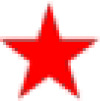
DTX l	NM_004416:exon1:c.G99:p.E33D	40.30%	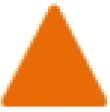
UBE2 A	NM_003336:exon6:c.G343A:p.El l5K	90.59%	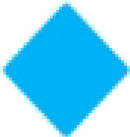
BTGl	NM_00173 l:exon2:c.A2 17C:p.N73H	44.56%	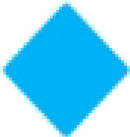
DTX l	NM_004416:exon l:c.Al l9G:p.Y40C	43.04%	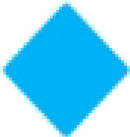
SOCSI	NM_003745:3xon2:c.C44T:p.T151	14.80%	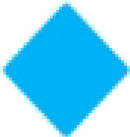
PCLO	NM_033026 :exon3:c.G244 1C:p.S814T rs2877	99.24%	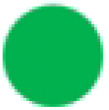
CCND3	NM_001760:exon5:c.T775G:p.S259A rsl 05 1130	99. l0%	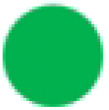
PCLO	NM_033026:EXON5:c.G8410a:p.A2804T rs976714	98.76%	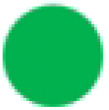
IDSTIHlC	NM_0053 19:exon l:c.C53T:p.A l 8V rs2230653	95.23%	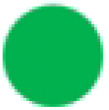
PRDMl	NM_00 1198:exon2:c.G220A :p.G74S rs2185379	49.07%	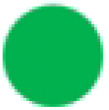
TNFRSF14	NM_003820:exon l:c.A50G :p.K l 7R rs4870	53.67%	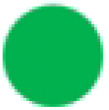
EZH2	NM_004456:exon6:c.G553C :p.D l 85H rs2302427	33.95%	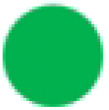

TP53 was a primary variation, indicating a poor prognosis (December, 2021). SNP, single nucleotide polymorphism; NGS, next-generation sequence.
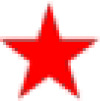
Primary variation 
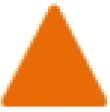
Secondary variation 
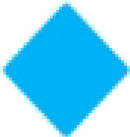
Tertiary variation 
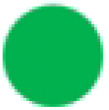
SNP.

**Figure 3:**
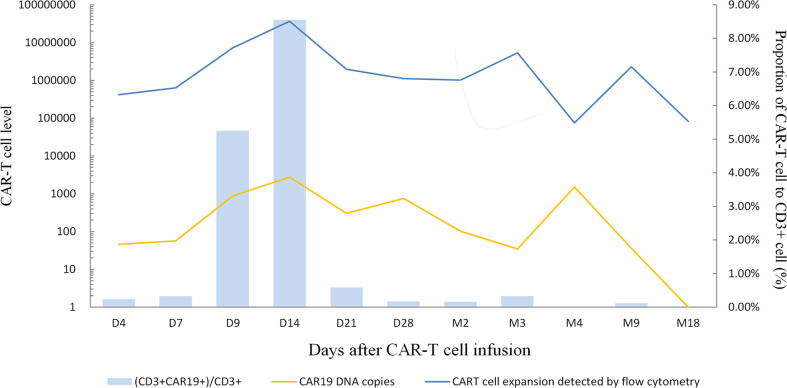
CAR19 DNA copies, CART cell expansion levels detected by flow cytometry and the proportion of CAR-T cell to CD3+ cell during treatment. The peak levels appeared on day 14 after CAR-T cell infusion, indicating CAR19 DNA copies were 2.79×103 copies/μg, CAR-T cells expansion detected by flow cytometry was 3.64×107/L and CD3+CAR19+/CD3+ T-cell was 8.55%.

The patient’s disease was refractory to two previous lines of treatment and kept progressing. Therefore, she was enrolled in the clinical trial of CD19 CAR-T cell therapy in June 2020. Headache, dizziness, and unsteady walking presented on the fifth postoperative day, and the patient had a poor response to mannitol for dehydration and intracranial pressure reduction. Methotrexate was given at 5 g for CNS infiltration on day -12. Fludarabine (45.25 mg/m^2^, days -3 to -1) and cyclophosphamide (925 mg/m^2^, days -2 to -1) were administered daily for lymphodepletion. Anti-CD19 CAR-T cells were infused as 1.0×10^6^cells/kg on day 0. Previous symptoms of this CNS relapse were not resolved, and visual-field defect occurred on the first day after anti-CD19 CAR-T cell infusion. Meanwhile, the patient’s DNA copy level of the CD19 CAR-T cells in peripheral blood did not show obvious expansion, presenting as 4.6×10^1^ copies/μg on day 4 and 5.65×10^1^ copies/μg on day 7 (**Figure 3**). Neurological symptoms were not relieved and progressed on day 5 after a series of intracranial pressure reduction therapies, including administration of mannitol, glycerol fructose, and low-dose dexamethasone. However, the patient suffered a sharp increase in blood pressure at 168/88 mmHg and decreased heart rate at 47/min on day 8. The combination of BTK inhibitor (zanubrutinib; 160 mg) with CD19 CAR-T cell therapy was used to improve treatment efficacy (day 8). A week later, her elevated intracranial pressure (ICP) symptoms persisted with hypertension, heart rate reduction, intermittent dizziness, and headache with the support of dehydrating agents. DNA level of the CD19 CAR-T cells expanded slowly to 2.79×10^3^ copies/μ*g* on day 14. Therefore, a PD-1 inhibitor (tislelizumab; 200mg) was applied on day 15. The previous adverse events abated after a week without dehydrating agents, and the patient achieved partial response (PR) on day 28. Two months later, the patient was evaluated as CR by PET/CT; brain MRI; and neck, chest, and abdomen CT scan with significant clinical response (**Figures 2B, C**). The DNA expanded level of CD19 CAR-T cells increased but still remained low in the peripheral blood until 4 months after infusion.

After 5 months of CD19 CAR-T cell therapy combined with zanubrutinib and tislelizumab, the patient presented with menorrhagia and severe anemia. The patient received a red blood cell transfusion and responded poorly to hemostatic agents in the local hospital. The PET/CT examination revealed complete molecular response (CMR) for DLBCL post-therapy but abnormally high metabolism in uterus (SUV max, 13.5). Afterward, the patient underwent hysterosalpingectomy and was confirmed with grade II endometrial carcinoma. The following radiotherapy for endometrial cancer was delayed because of the Covid-19 epidemic. Approximately 18 months after CD19 CAR-T cell treatment, no recurrence of symptoms was noted as stringent CR according to our follow-up (**Figure 1**).

## Discussion

In the course of this case, the patient has failed previous multiline chemotherapy. This may be associated with TP53 mutation, which is always strongly associated with drug resistance and dismal prognosis as a negative indicator (11). Previous studies suggest CAR-T cells could potentially overcome the detrimental influence of TP53 expression (12). In addition, the disease aggressively progressed and rapidly relapsed with the longest interval shorter than 6 months. For DLBCL patients, the median survival time was less than one year if the disease recurrence interval was shorter than six months after remission and even worse in patients with CNS relapse (14). Thus, this patient had an extremely poor prognosis before CAR-T cell therapy. The administration of CAR-T cells was given only 1×10^6^/kg, considering BBB was damaged due to the CNS relapse, and CAR-T cells became more permeable. However, anti-CD19 CAR-T cells initially expanded slowly in peripheral blood after infusion, but it may contribute to low toxicity and fewer adverse events. The peak level of CAR-T cells appeared on Day 14 (CAR19 DNA copies: 2.79×10^3^ copies/μg; CAR-T cell expansion was detected by flow cytometry: 3.64×10^7^/L; CD3+CAR19+/CD3+ T-cell: 8.55%), far from the ideal functional concentration (**Figure 3**). IL-6 and TNF-α were detected only on days 7 and day 14 at normal levels. The hematological indexes, cytokines, ferritin, and C-reaction protein (CRP) levels as well as temperature all remained normal (**Supplementary Files**, **Figures S1**–**S4**). Persisting neurological symptoms indicated disease progression rather than cytokine release syndrome (CRS) or immune effector cell associated neurotoxicity syndrome (ICANS). To the best of our knowledge, the patient’s symptoms relieved rapidly after using tislelizumab. According to brain MRI, the patient’s intracranial masses were significantly reduced after 5 months of the combination treatment (**Figure 2C**). The mechanism of incorporating of BTK inhibitor and PD-1 antibody may promote CAR-T cells’ efficacy by improving the tumor microenvironment. This patient maintained great CR status with zanubrutinib and tislelizumab maintenance up to the latest follow-up (December 2021).

SCNSL has an extremely poor prognosis. Despite recent advances in treatment modalities, there is no standard and effective treatment guideline for SCNSL. Previously, a CD19 CAR-T cell therapy study emphasized a high complete remission rate and overall response rate in lymphoma patients, ranging from 40% to 60% and from 50% to 80%, respectively. However, a high relapse rate and failure risk remained elusive (15). In this case, we applied the second generation CARs containing the 4-1BB co-stimulatory signal for therapy. In most cases, CAR-T cells with 4-1BB domain possessed mild reactions with fewer ICANs and longer persistence for more than 6 months (16, 17). During R/R NHL treatment, CD19 CAR-T cells with 4-1BB co-stimulatory domain had superior safety profiles and fewer adverse events but was less effective to the disease compared with CD28 as a co-stimulatory molecule in numerous studies (18–21). Recently, a novel agent designed IM19 with 4-1BB-based co-stimulatory signal possessed durable antitumor activity to improve clinical efficacy (22).

Few reports are available on CAR-T cell therapy for SCNSL because most R/R B-cell lymphoma patients with CNS infiltration are excluded from CAR-T clinical trials. An immunosuppressive microenvironment could contribute to the escape of immune surveillance. The immunosuppressive factors, such as IL-10, TGF-β, and IDO, would possibly inhibit the activation of CAR-T cells and decrease the therapeutic effect (17). However, the immune checkpoint pathway plays a critical role in inhibitory signals to escape immune surveillance, especially the PD-1/PD-1 ligand (PD-L1) signaling pathway. It triggers T cell exhaustion and tumor tolerance. The tumor inhibitory microenvironment could be improved by blocking the PD-1/PD-L1 signaling pathway, leading to an increased T cell number and promoting antitumor efficacy. PD-1 was highly expressed in CAR-T cells, which caused weak antitumor immune response (6, 23, 24). The anti-PD-1 agent could be applied before CAR-T cell therapy or as a single agent for those extranodal relapsed DLBCL patients. PD-1 antibody enhanced CAR-T cells’ function and prolonged their therapeutic effect by improving the immune microenvironment (25–29).

In terms of R/R B-cell NHL management, BTK inhibitors provide an opportunity to start a chemotherapy-free era as novel agents. Considerable research has been devoted to expanding its application for antitumor effects as targeted agents or combined with chemotherapy and immunotherapy in the last decades. Zanubrutinib is a next-generation BTK inhibitor that exhibits highly potent and less off-target toxicity (30). One pooled analysis of two clinical trials (BGB-3111-AU-003 and BGB-3111-206) revealed favorable efficacy of zanubrutinib monotherapy in R/R mantle cell lymphoma (MCL), produced an objective response rate (ORR) of 84.8% and a CR rate of 62.5% (31). In the CNS microenvironment, a hypothesis indicated that chronic antigen presentation and BCR stimulation could possibly promote BTK dependence. Zanubrutinib has superior target effects by blocking several essential molecular pathways, including BCR signaling, BTK or B-lymphocyte kinase, Toll-like receptor (TLR) signaling and downregulating exhaustion markers, such as PD-1, TIM-3, and LAG-3 (30). Zanubrutinib could cross the BBB to exert its effect, alleviating Bing–Neel syndrome with CNS lymphoplasmacytic cell infiltration among Waldenström macroglobulinemia (WM) patients and successfully applied to refractory PCNSL case (32, 33). Notably, emerging evidence has identified that zanubrutinib could modulate the immune system by inhibiting interleukin-2-inducible T-cell kinase (ITK) in T cells, which reduces T cell differentiation and shifting the balance of Th1/Th2 cells or enriches Th17 cell subsets. BTK inhibitor possesses complex interaction with CAR-T cells, which might optimize its proliferation and high tumor clearance effect under multiple preclinical trials. Narendranath et al. proposed the idea that administration of a BTK inhibitor before T cell collection could promote the level of IL-2 and IFNγ, which are associated with high self-renewal ability and production efficacy, respectively, as well as stronger cytotoxicity. Meanwhile, a case reported by Weiguo Zhu et al. considered dual inhibition of HDAC and BTK resulting in long-term remission with R/R DLBCL patients after failure of CAR-T cell therapy with TP53 mutation, which suggests underlying synergistic mechanism between BTK inhibitor and CAR-T cell therapy (7). Furthermore, BTK inhibitor enabled the reduction of PD-1 and cytotoxic T lymphocyte-associated protein 4 (CTLA-4) to overturn the exhausted T cell phenotype. Consequently, combining PD-1 antibody and BTK inhibitor was demonstrated to possess a stronger synergistic antitumor reaction than any single one of them with a manageable safety profile, resulting in 40% objective response rate (ORR) among GCB-DLBCL patients (30). However, the combination of PD-1 antibody or BTK inhibitor requires a novel approach to tackle their drug resistance and immune regulations such as CAR-T cell therapy, which could lead to a striking response (30).

## Concluding remarks

This case report study describes a refractory DLBCL patient who developed CNS relapse and was successfully treated with anti-CD19 CAR-T cell therapy plus BTK inhibitor and PD-1 antibody. The therapy reversed the patient’s dangerous condition and led to remarkable response without any ICANS. This patient sustained CR for more than 18 months without any adverse event by continuously taking zanubrutinib and tislelizumab. However, limitations still remain in our study. The BTK inhibitor and PD-1 antibody showed synergistic effects to CAR-T cell therapy, but we cannot figure out their mechanisms of action, respectively. Further research efforts should focus on the potential treatment efficacy of CAR-T cell therapy with multimodality adjuvant protocol. Despite limitations, this treatment scheme provided an impetus and inspired-future strategies for similar disease. The successful experience with this case warrants further clinical studies.

## Data availability statement

The original contributions presented in the study are included in the article/**Supplementary Material**. Further inquiries can be directed to the corresponding author.

## Ethics statement

The studies involving human participants were reviewed and approved by the Institutional Ethics Committee of the Fourth Hospital of Hebei Medical University. The patients/participants provided their written informed consent to participate in this study. Written informed consent was obtained from the individual(s) for the publication of any potentially identifiable images or data included in this article.

## Author contributions

RL was the physician-in-charge. WZ, LL designed the clinical protocol and wrote the manuscript. SY, WJL, LW and WL were the clinicians who participated in the treatment of the patient. HZ analyzed data. All authors contributed to the article and approved the submitted version.

## Funding

This study was funded by the 2021 Hebei Provincial Department of Finance government-funded clinical medicine talent training project: Application of human CD19 CAR-T cells in recurrent, refractory B-cell lymphoma to L.L.H.

## Conflict of interest

The authors declare that the research was conducted in the absence of any commercial or financial relationships that could be construed as a potential conflict of interest.

## Publisher’s note

All claims expressed in this article are solely those of the authors and do not necessarily represent those of their affiliated organizations, or those of the publisher, the editors and the reviewers. Any product that may be evaluated in this article, or claim that may be made by its manufacturer, is not guaranteed or endorsed by the publisher.

## Supplementary material

Supplementary Figure 1 Hematological indexes remained normal during the treatment process.

Supplementary Figure 2 Changes in Ferritin and C-reaction protein (CRP) levels before and after CAR-T cell infusion.

Supplementary Figure 3 Serum levels of cytokines were monitored after CAR-T cell infusions. Elevated serum of IL-18 was detected on day 21 and 28, respectively. IL-17A and MCP-1 reached peak level on day 7 and day 28, respectively.

Supplementary Figure 4 The temperature reached 37.8°C on the eighth day, accompanied by severe symptoms of intracranial hypertension, then recovered quickly and remained normal for the rest of the time.
