# Supplementary figures and images for: Case report: CD19-directed CAR-T cell therapy combined with BTK inhibitor and PD-1 antibody against secondary central nervous system lymphoma
Source: Front Immunol. 2022 Oct 5;13:983934. doi: 10.3389/fimmu.2022.983934 (PMC9581047; doi:10.3389/fimmu.2022.983934)

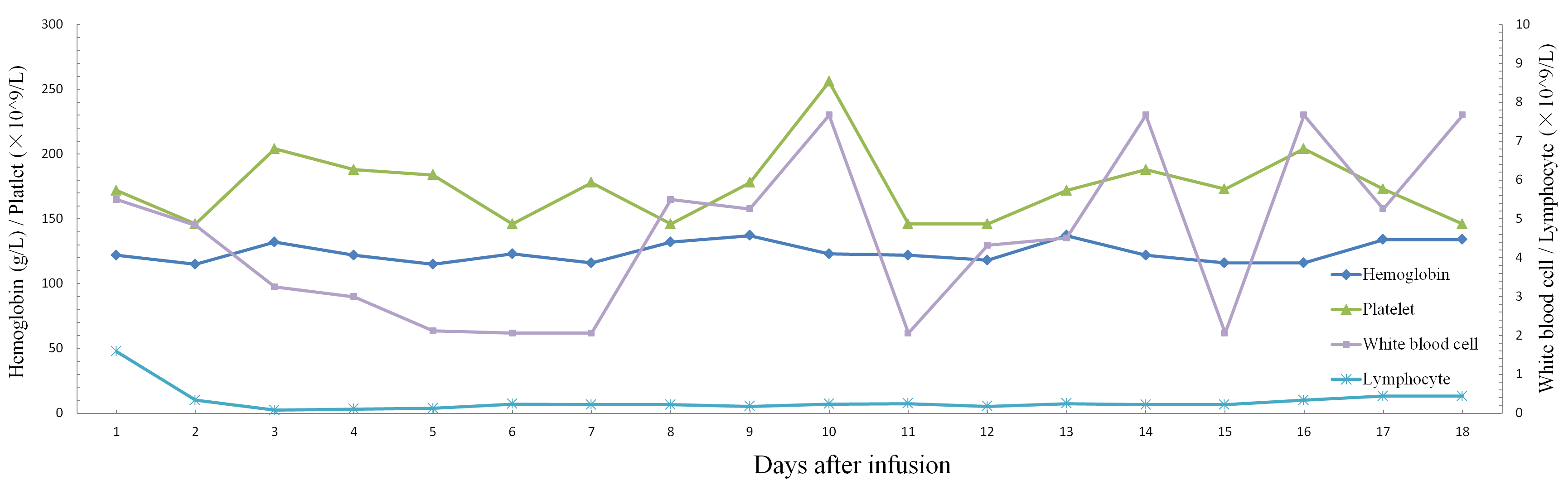

Supplement: Supplementary Figure 1 — Hematological indexes remained normal during the treatment process. [file DataSheet_1.zip › Additional Figures/Figure S1.png]

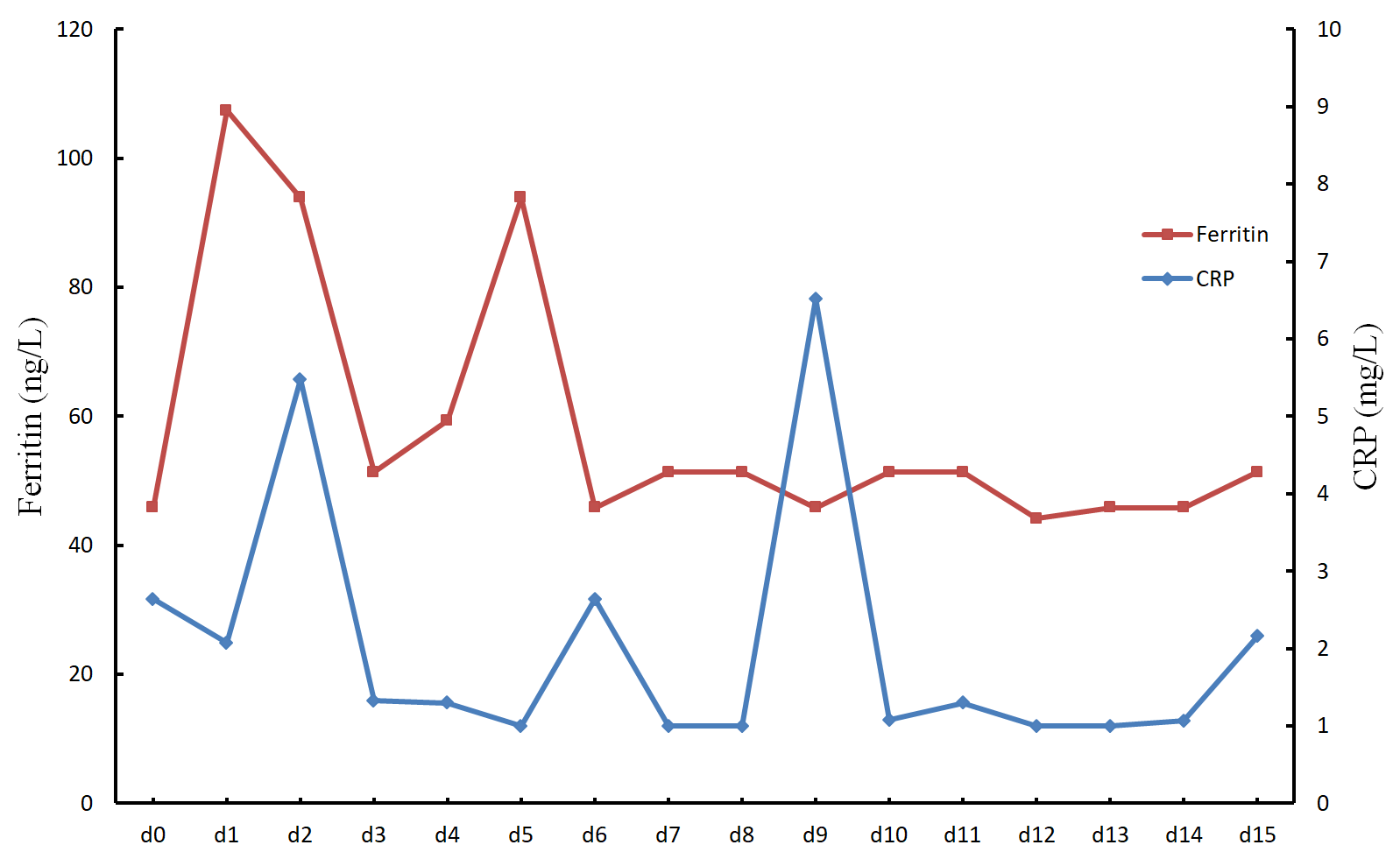

Supplement: Supplementary Figure 1 — Hematological indexes remained normal during the treatment process. [file DataSheet_1.zip › Additional Figures/Figure S2.png]

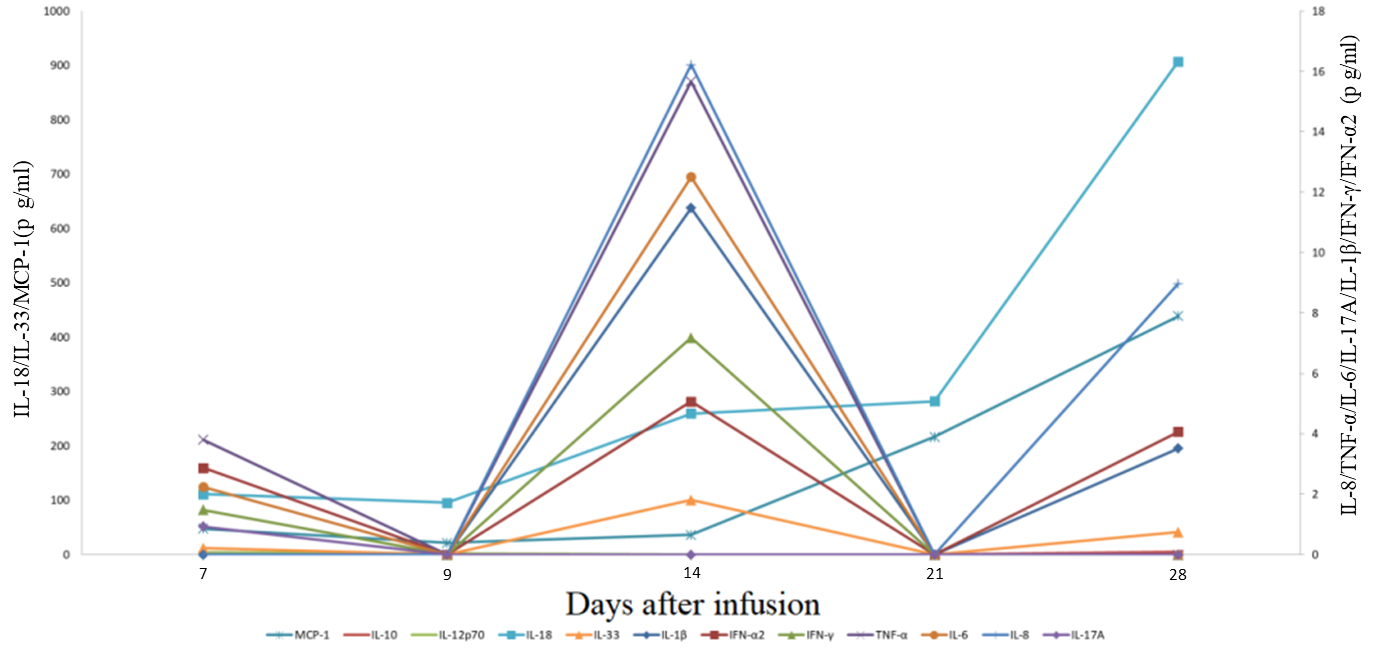

Supplement: Supplementary Figure 1 — Hematological indexes remained normal during the treatment process. [file DataSheet_1.zip › Additional Figures/Figure S3.png]

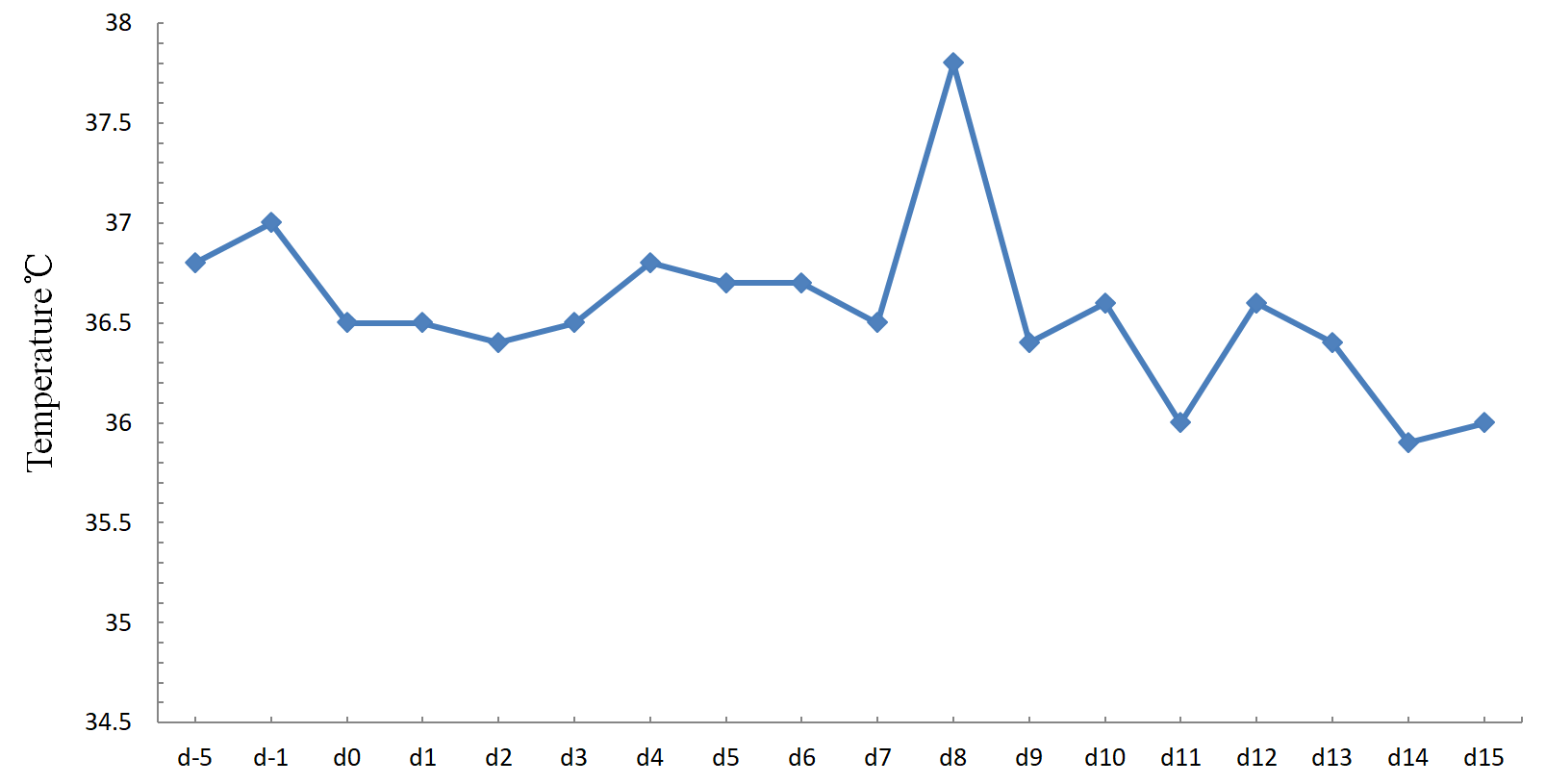

Supplement: Supplementary Figure 1 — Hematological indexes remained normal during the treatment process. [file DataSheet_1.zip › Additional Figures/Figure S4.png]
